# Vaccine in Gonococcal Affections

**Published:** 1909-12-04

**Authors:** 


### Medicine.

### VACCINE IN GONOCOCCAL AFFECTIONS.

One of the largest collections of consecutive cases of gonococcal affections treated by the vaccine method, forms the subject of an analytical paper by Dr. Eyre and Dr. Stewart in the *Lancet*.* Their summary of conclusions may be arranged under three main headings, according as the cases dealt with were affected by acute gonorrhœa, chronic gonorrhœa without complications, and chronic gonorrhœa with complications, respectively.

As regards acute gonorrhœa they hold that:—

1. Gonococcus vaccine is markedly toxic and exerts a profound influence over the disease.

2. For routine work—hospital out-patients, etc.—vaccine treatment is not devoid of danger, and requires the exercise of considerable caution.

3. A stock vaccine, comprising a dozen different strains, gives results only slightly inferior to those observed when using a vaccine prepared from the patient's own organism. This is not the rule in most other diseases.

4. Small doses, repeated at short intervals, are more effective than large doses at lengthened intervals.

5. Small doses of vaccine—from 1,000,000 to 10,000,000—are safer and more satisfactory than the large doses—from 50,000,000 to 100,000,000—which are often prescribed.

6. After an injection of from 500.000 to 2,000,000 the negative phase is either absent or extremely transient.

7. An inoculation of from 5,000,000 to 10,000,000 causes a negative phase of usually not longer than forty-eight hours' duration, followed by a positive phase of from three to five days.

8. Vaccines in small doses serve the double purpose of raising and steadying the opsonic index. A steady index just above normal is found to be the most favourable condition for rapid recovery.

As regards uncomplicated chronic gonorrhœa they hold that:—

1. Where the gonococcus has ceased to be the infecting organism, these cases are on a par with other chronic inflammatory states, but are frequently more difficult to cure owing to environment and local conditions.

2. Chronic cases where the gonococcus is the sole infecting organism have a better prognosis from the point of view of treatment by vaccine than a mixed infection or one of staphylococcus only.

As regards chronic gonorrhœa with complications, they hold that:—

1. The estimation of the opsonic index is helpful to diagnosis, and is a useful means of determining approximately the opsonic state of the blood. Chronic gonococcus infections, however, present clinical features, which themselves afford valuable indications during the course of vaccine treatment.

2. Where the gonococcus alone is the infecting organism, if the opsonic index cannot be obtained as frequently as is desirable, routine injections of from 1,000,000 to 2,000,000 doses every three to five days are safe and satisfactory; a lapse of five to seven days should be allowed after doses of 5,000,000, and an interval of eight to ten days after an inoculation of 10,000,000. Larger doses than these are seldom desirable.

3. Treatment by small and gradually increasing doses at frequent intervals should at all times be adhered to; the use of large doses is even more dangerous than in acute cases, and may be followed by disastrous consequences.

4. In orchitis small doses of vaccine quickly relieve pain and cause a more rapid abatement of symptoms than obtains by the usual routine treatment alone.

5. In iritis the severe pain, which is a marked and obstinate feature, is relieved in forty-eight hours after an injection, and disappears in from three to four days; cure is much hastened.

6. In arthritis the treatment is of considerable value.

* *Lancet*, 1909, ii., pp. 76 to 81.

